# The detrimental impact of seminal plasma on the quality of cryopreserved sperm in goat bucks: freezing–thawing as the most harmful step

**DOI:** 10.3389/fvets.2025.1627878

**Published:** 2025-08-04

**Authors:** Inés Carolina Esteve, María Lorena Mocé, Ernesto Angel Gómez, Rosario Martín-Montalbán, Andrea Martínez-Climent, Eva Mocé

**Affiliations:** ^1^Centro de Investigación y Tecnología Animal (CITA), Instituto Valenciano de Investigaciones Agrarias (IVIA), Segorbe, Spain; ^2^Unidad Asociada UCH-CEU- IVIAValencia, Spain; ^3^Department of Animal Production and Health, Veterinary Public Health and Food Science and Technology (PASAPTA), Facultad de Veterinaria, Universidad Cardenal Herrera-CEU (UCH-CEU), CEU Universities, Alfara del Patriarca, Spain

**Keywords:** semen, cryopreservation, caprine, cytometry, seminal plasma

## Abstract

**Introduction:**

Seminal plasma (SP) from goat bucks must be removed before freezing for obtaining sperm surviving the cryopreservation process if egg yolk-or skimmed milk (SM)-based extenders are used.

**Methods:**

We studied the effect of SP on goat sperm quality in the steps of the freezing process (F: after centrifugation and addition of SM extender; R: after refrigeration to 4°C; G: after SM-glycerol extender addition; E: after equilibration; T: after freezing–thawing). Ejaculates (*n* = 21) from Murciano-Granadina goat bucks were split into two samples: one was processed with SP (SP+) and the other without SP (SP−). Sperm quality was evaluated in all the steps (F, R, G, E, and T).

**Results:**

Refrigeration induced damage to the samples SP+ but not to the samples SP−. The worsening (*p* < 0.05) of the parameters indicates that SP is especially noxious in this step for the acrosome, plasma membrane, and sperm mitochondria. The addition of glycerol exerted negative effects in both treatments, while the equilibration phase did not add further damage to the sperm. The freezing–thawing was the step that provoked the most damage in both treatments. After thawing, SP− samples retained around 50% of the values they exhibited after equilibration for total motile sperm or live sperm with intact acrosome, while these values dropped to 10% in SP+ samples.

**Discussion:**

The detrimental effect of SP is evident from the refrigeration step, but the most harmful is the freezing–thawing step. Meanwhile, the equilibration step does not affect sperm quality.

## 1 Introduction

Artificial insemination (AI) is a fundamental tool in goat breeding programs (1) that enables spreading the genetic response to the commercial herds, testing candidate bucks, and connecting herds through relatives (2). Although AI can be performed with cryopreserved sperm, refrigerated semen doses are preferred in dairy goat farms due to their higher fertilizing potential (3). Nevertheless, cryopreserved sperm is a key pillar for gene conservation, maintenance of endangered species and breeds, and distribution of genes without limitation of distance and time (4).

Ejaculates are a mixture of spermatozoa and seminal plasma (SP). This SP consists of a mix of fluids from various male accessory glands and from the cauda epididymis (5) and is composed of amino acids, proteins, ions, electrolytes, metabolites like nucleosides, lipids, monosaccharides, minerals, enzymes, and steroid hormones (6). This secretion facilitates the survival of spermatozoa both *in vitro* and *in vivo* (7), participates in different cell signalling processes, and provides energy to sperm, due to the variability of sugars and lipids it contains. Furthermore, SP factors are also implied in the regulation of capacitation, stimulation of motility, semen storage in the female tract, and modulation of the female’s immune system for sperm tolerance (7).

Despite its important roles in sperm physiology and endometrial and embryo gene expression modulation (8), the presence of SP in goats is deleterious for frozen sperm (9) to the point that SP removal is mandatory for obtaining sperm surviving the freezing–thawing processes. Diluents commonly used for freezing goat sperm contain skimmed milk (SM) or egg yolk, and some of their components are substrates for enzymes (BUSgp60 and egg yolk-coagulating enzyme) secreted by the bulbourethral gland. The enzymatic reactions triggered by these lipases release metabolites that are toxic for the sperm (i.e., oleic acid coming from the degradation of residual triglycerides from SM), that induce the acrosome reaction and subsequent cell death when spermatozoa are incubated in milk medium at 37°C (10, 11). In contrast, the SP is not detrimental for liquid stored goat buck sperm (10), although the diluents are those used for freezing, except glycerol. Indeed, refrigerated goat buck seminal doses include SP, and fertility remains unaffected when these doses are used on the same day they are produced (10).

Although it is known that SP is detrimental for goat sperm freezing, it remains to be elucidated at which point of the freezing protocol the damage occurs. Thus, this study aimed to determine the effect of SP on goat buck sperm quality in each of the steps of the semen freezing process (fresh, refrigeration, glycerol addition, equilibration, freezing and thawing). Understanding the mechanisms of damage during the cryopreservation protocol could lead to innovative solutions that improve the overall quality and reproductive performance of frozen–thawed sperm. This research is particularly relevant to breeding programs, where performing artificial inseminations with cryopreserved sperm can enhance genetic selection by increasing the use of catalog males in commercial farms and supporting the conservation of genetic diversity.

## 2 Materials and methods

### 2.1 Materials and preparation of diluents

All chemicals were reagent grade and purchased from Sigma-Aldrich (Madrid, Spain), except for propidium iodide (PI) and Mitotracker deep red FM, which were purchased from Invitrogen (Barcelona, Spain). The diluents Tris-citrate-glucose (TCG) and TCG supplemented with bovine serum albumin (BSA; 0.3%; w/v) were used to perform the analyses for sperm quality evaluation following the protocols described in Mocé et al. (3). Two skimmed milk-based (SM) diluents were used for freezing. The first diluent (SM1) was made with skimmed milk (Central Lechera Asturiana; Oviedo, Spain) and 0.2% (w/v) of D (+)- glucose. The second diluent (SM2) was made with SM1, adding 28% glycerol (v/v). The sodium chloride solution (0.9%; w/v) was used for sperm concentration determination.

### 2.2 Animals and semen extraction

This study was carried out in the Centro de Tecnología Animal, Instituto Valenciano de Investigaciones Agrarias (CITA-IVIA; Segorbe, Castellón, Spain), located in the North-East of Spain (39°52′N, 0°30′W). Seven adult Murciano-Granadina goat bucks were used as semen donors. They were housed in pens with access to water and straw ad libitum. Moreover, they were supplied with 1 kg/day of concentrated feed (17% crude protein, 11.6% crude fiber, and 4.5% crude fat) per male. The protocols for semen collection, care, and animal housing complied with European regulations for the care and use of animals for scientific purposes (12). According to legislation, semen collection with an artificial vagina is considered as a routine husbandry practice and, for this reason, does not require ethical approval. The males were not considered experimental animals, and no special ethical permission was required.

Semen was extracted in the morning with artificial vagina following the protocol described in Silvestre et al. (13). These animals were used to serving the artificial vagina and semen was regularly collected (at least once/week) throughout the year. In total, 21 ejaculates collected during the reproductive season (from July to November) were processed (three ejaculates/male). Tubes containing the ejaculates were immersed in a water bath at 25°C until used. Volume and sperm concentration were measured according to the protocols described in Konyali et al. (14).

### 2.3 Freezing–thawing protocol

Each ejaculate was split into two aliquots: one of them was frozen with SP (SP+), and in the other one, the SP was removed before freezing (SP−). The samples that retained the SP were kept at 22°C until the SP was removed from the other samples. The SP was removed as described in Konyali (15). Briefly, each sample was diluted with TCG up to 10 mL, centrifuged for 15 min at 500*g* at room temperature (~22°C), and the supernatant was discarded. Then, the pellet was resuspended in 10 mL of TCG and centrifuged again. After this, the supernatant was removed, and the pellet was homogenized in the remaining TCG extender. Then, the volume and concentration were again evaluated (obtaining an average of 0.73 mL and 2,878 × 10^6^ sperm/mL).

The sperm concentration in each sample (with or without SP) was adjusted to 667 × 10^6^ sperm/mL with SM1 at 22°C. Then, the samples were refrigerated from 20°C to 4°C in a programmable water bath (Julabo GmbH, Seelbach, Germany) for 90 min at a rate of −0.18°C/min (3).

After refrigeration, the samples were transferred to a cold room (set at 4°C) to continue with the protocol. First, a cocktail of antibiotics was added (comprised of 300 μg of spectinomycin, 250 μg of gentamycin, 150 μg of lincomycin, and 50 μg of tylosin per mL of frozen semen; Minitub Ibérica S.L., Tarragona, Spain), according to the recommendations from the OIE (16). Then, precooled SM2 was added (dilution 3:1; v/v) to the samples to obtain final concentrations of 500 × 10^6^ sperm/mL and 7% glycerol (v/v). To avoid osmotic shock, the volume was divided into three parts that were added at 10 min intervals to the samples. After the last addition, the samples were equilibrated for 90 min at 4°C, during which the semen was loaded into 0.25 mL plastic straws (IMV Technologies, L’Aigle, France) that were sealed with polyvinyl alcohol (PVA, IMV Technologies, L’Aigle, France). After equilibration the straws were frozen in a programmable freezer (Minidigitcool, IMV Technologies, L’Aigle, France) at the following rates: −4°C/min from 4 to −5°C, −25°C/min from −5 to −110°C and −35°C/min from −110 to −140°C and later, they were plunged into liquid nitrogen for storage.

The straws were thawed by immersion in a water bath at 37.5°C for 30 s.

To perform the analyses in the laboratory, 40 μL aliquots were taken at these points of the freezing protocol: fresh (after SM1 addition), refrigeration (after chilling to 4°C), glycerol addition (after the addition of SM2), equilibration (once the 90 min period of equilibration with glycerol concluded) and thawing (after freezing–thawing).

### 2.4 Sperm quality evaluation

To determine the seminal quality, sperm motility and sperm plasma membrane integrity (PMI), acrosomal integrity and mitochondrial functionality were evaluated at each point of the freezing–thawing protocol previously described. These analyses were performed according to the protocols described by Mocé et al. (17). All the analyses were performed at room temperature (22°C). The samples were first diluted to a concentration of 30 × 10^6^ sperm/mL with TCG. This dilution with TCG was progressively performed in the samples with glycerol (after SM2 addition, equilibration, and freezing–thawing). For this, a previous 1:3 dilution (v/v) was performed with TCG before the final dilution to 30 × 10^6^ sperm/mL.

Motility was evaluated in samples adjusted to 6 × 10^6^ sperm/mL with TCG-BSA and incubated at 37°C for 10 min before analysis following the protocols described in Mocé et al. (17). Briefly, these analyses were performed with a 10x negative phase contrast objective under a Nikon Eclipse microscope (IZASA, Barcelona, Spain). Subsamples of 7.5 μL were placed inside a Makler chamber (Counting Chamber Makler, Sefi-Medical Instruments, Haifa, Israel) prewarmed at 37°C on a thermal plate. The data from a minimum of 200 sperm from three different fields were collected. Individual sperm tracks were visually assessed to eliminate possible debris and wrong tracks. The quantity and quality of movement were determined in a CASA system (ISAS, version 1.0.17, Proiser, Valencia, Spain) that operated at 30 video frames per second (30 Hz). The particle area was set at 15–70 μm, and the search radius at 12 μm. The following variables were obtained: percentages of total (TM; %) and progressively motile (PM; %) sperm, average path velocity (VAP; μm/s), curvilinear velocity (VCL; μm/s), straight line velocity (VSL; μm/s), straightness index (STR; VSL/VAP × 100; %), linearity (LIN; VSL/VCL × 100; %), wobble (WOB; VAP/VCL × 100; %), amplitude of the lateral movement of the head (ALH; μm) and beat cross frequency (BCF; Hz). The spermatozoa were classified as motile if their VAP > 10 μm/s and PM if they presented VAP > 75 μm/s and STR ≥ 80%.

Acrosomal and plasma membrane integrity and mitochondrial functionality were evaluated with flow cytometry following the protocol described in Mocé et al. (17). These structures were evaluated with fluorescent stains and a CytoFLEX S flow cytometer (Beckman Coulter Life Sciences, L’Hospitalet de Llobregat, Barcelona, Spain) equipped with three lasers (a 50-mW 488-nm blue laser, a 50-mW 638-nm red diode laser and an 80-mW 405-nm violet laser) and the CytExpert software (Beckman Coulter Life Sciences, L’Hospitalet de Llobregat, Barcelona, Spain). CytoFlex Daily QC fluorospheres were used to verify the flow cytometer’s optical alignment and fluidics system according to the manufacturer’s instructions. Samples were treated by quadruple staining with Hoechst 33342, propidium iodide (PI), fluorescein isothiocyanate-conjugated peanut agglutinin (FITC-PNA), and Mitotracker deep red FM. Samples were stained for the flow cytometric analysis by transferring 0.1 mL aliquots with 3 × 10^6^ sperm to tubes with 25 μL of TCG diluent, 5 μL of Hoechst (0.1 mg/mL stock solution in Milli-Q water), and 0.25 μL of Mitotracker (25 μM stock solution in DMSO). Samples were incubated for 20 min at room temperature in the dark. Then, a solution containing 25 μL of TCG diluent with 0.25 μL of PI (1 mg/mL stock solution in Milli-Q water) and 0.5 μL of FITC-PNA (1 mg/mL stock solution in Milli-Q water) was added to each sample. Samples were incubated for another 10 min before being diluted with 0.40 mL of TCG and analyzed. Hoechst was excited with the violet laser, and its fluorescence was detected using a 450/45 nm avalanche photodiode (APD). Mitotracker was excited with the red laser, and its fluorescence was detected employing a 660/20 nm APD. PI and FITC-PNA were excited with the blue laser. The red fluorescence of PI was detected using 690/50 nm APD, and the green fluorescence of FITC-PNA was detected using 525/40 nm APD. Then 50,000 events per sample were analyzed. The compensation between PI-FITC-PNA was 0.93 and 1.39% between PI-Mitotracker. Non-DNA-containing events (Hoechst-negative) were excluded, and the sperm population was gated based on the expected forward and side scatter signals.

PI penetrated non-viable cells to distinguish three populations: live (LIVE; PI-; plasma membrane intact sperm; %), and plasma membrane damaged sperm (PI+; %) distinguishing two populations, one with high PI fluorescence intensity and another one with low PI fluorescence intensity (APOPTOTIC; apoptotic sperm; %). Only the sperm with damaged acrosomes stained with FITC-PNA and as a result two populations were distinguished: acrosome-reacted sperm (AR; FITC-PNA+; %) and acrosome-intact sperm (AI; FITC-PNA-; %). Finally, all the sperm were stained with Mitotracker, and two populations were distinguished: one with low intensity corresponding to the sperm with low mitochondrial membrane potential (MMP; %) and another with high intensity corresponding to the sperm with high MMP (MITOK; %).

First, spermatozoa were categorized on a chart according to stains PI and FITC-PNA as dead acrosome intact sperm (MAI, %; PI+/FITC-PNA-), dead acrosome reacted sperm (MAR, %; PI+/FITC-PNA+), live acrosome intact sperm (VAI, %; PI-/FITC-PNA-), live acrosome reacted sperm (VAR, %; PI-/FITC-PNA+). Then, PI and Mitotracker were also plotted on another chart, and four sperm populations were obtained: dead with low mitochondrial membrane potential (DMM, %; PI+/Mitotracker-), dead with high mitochondrial membrane potential, (DMB, %; PI+/Mitotracker+), live with low mitochondrial membrane potential, (LMM, %; PI−/Mitotracker-), live with high mitochondrial membrane potential, (LMB, %; PI−/Mitotracker+).

The results considered the following variables: TM, PM, VSL, VAP, VCL, LIN, STR, WOB, ALH, BCF, AR, LIVE, APOPTOTIC, VAI, LMB, and MITOK.

In addition, to compare the relative success of the process, new variables were generated for TM, PM, VAI, and acrosome-intact sperm (AIS = 100−AR). The relative changes between different process steps were calculated to generate the new variables (rTM, rPM, rVAI, and rAIS). For example, for comparing the relative success of all the processes (fresh vs. frozen–thawed) on TM, the variable rTM was calculated as (TM frozen–thawed/TM fresh) × 100. For comparing the relative success of the last step of the process (equilibrated vs. frozen–thawed) on TM, the variable rTM was calculated as (TM frozen–thawed/TM equilibrated) × 100.

### 2.5 Statistical analyses

First, the variables considered for the experiment were subjected to Kolmogorov–Smirnov tests to confirm if the data followed a normal distribution so that they could be later analyzed with parametric tests. The following variables were confirmed to be normally distributed: LIVE, LMB, VAI, VCL, VSL, VAP, BCF, and ALH. The remaining variables (TM, rTM, PM, rPM, LIN, STR, WOB, AR, APOPTOTIC, MITOK, rVAI, and rAIS) were transformed following arcsine transformation, but results are reported in the original scale after retransforming them with the sine function.

All the variables were analyzed by fitting the model:


$$Y_{\text{ijklm}}=\mu +{{\text{treatment}}_{i}}^{*}{\text{step}}_{j}+{\text{session}}_{k}+{\text{male}}_{l}+{{\text{session}}_{k}}^{*}{\text{male}}_{l}+e_{\text{ijklm}}$$

Where treatment by step was a fixed effect with ten levels (presence of SP and step fresh, 1; presence of SP and step refrigerated, 2; presence of SP and step glycerol, 3; presence of SP and step equilibrated, 4; presence of SP and step thawing, 5; absence of SP and step fresh, 6; absence of SP and step refrigerated, 7; absence of SP and step glycerol, 8; absence of SP and step equilibrated, 9; absence of SP and step thawing, 10), session was the random effect of the session, male was the random effect of the male and session*male was the interaction of both random effects and e_ijklm_ was the random residual effect. One-way analysis of variance (ANOVA) was used, followed by Tukey’s *post hoc* tests. Statistical analyses were run using SPSS^®^ 27.0 (IBM Corporation, New York, NY, USA). The level of significance was set at *p* < 0.05 in all tests.

## 3 Results

**Figure 1:**
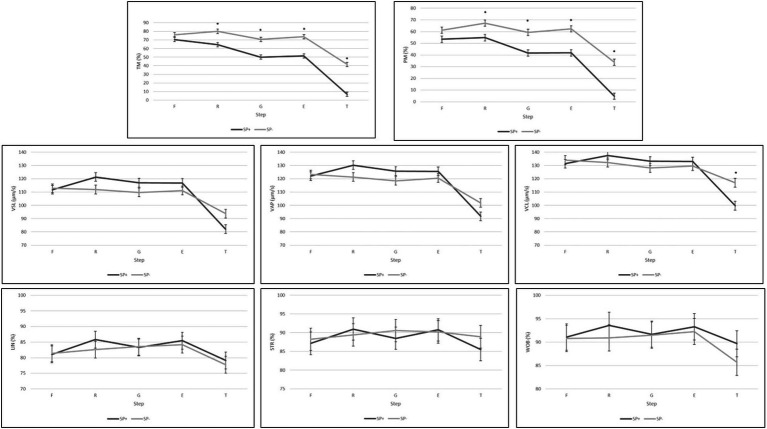
Goat bucks’ sperm quality (motility variables, *n* = 21) in each step of a freezing protocol when samples were processed in the presence (SP+) or in the absence (SP−) of SP. F, fresh (after centrifugation and addition of the first diluent); R, refrigerated (after reaching 4°C); G, glycerol (after addition of the extender containing glycerol); E, equilibrated (after 90 min of equilibration with glycerol at 4°C); T, thawed (after freezing–thawing); TM, total motile sperm (%); PM, progressive motile sperm (%); VSL, straight line velocity (μm/s); VAP, average path velocity (μm/s); VCL, curvilinear velocity (μm/s); LIN, linearity (%); STR, straightness index (%); WOB, wobble (%); *within a step, indicates significant differences between treatments (*p* < 0.05). All values are represented as least square means ± SE.

Figure 1 depicts the results for the motility variables comparing treatments (presence or absence of SP) in each of the steps of the freezing protocol. Significant differences were observed between treatments for TM and PM since the refrigeration step, exhibiting the SP− samples higher values (*p* < 0.05) than the samples SP+. For VCL significant differences (*p* < 0.05) were only observed between treatments after thawing. The values were lower in the thawing step for the samples SP+ (99.68 μm/s vs. 117 μm/s for SP+ and SP−, respectively). Values for VAP, VSL, WOB, STR, and LIN were similar for both treatments.

**Figure 2:**
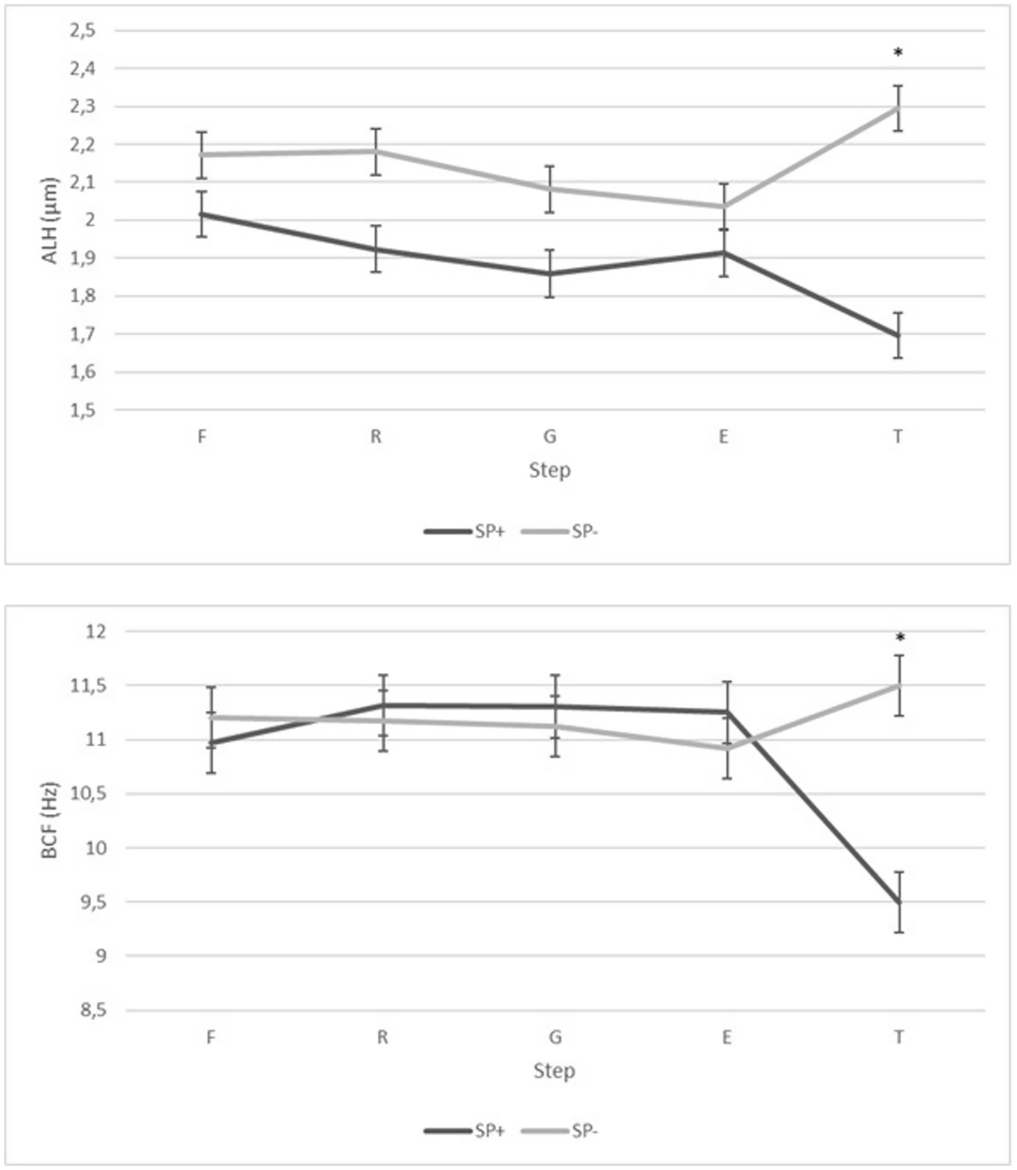
Goat bucks’ sperm quality (ALH and BCF, *n* = 21) in each step of a freezing protocol when samples were processed in the presence (SP+) or the absence (SP−) of SP. F, fresh (after centrifugation and addition of the first diluent); R, refrigerated (after reaching 4°C); G, glycerol (after addition of the extender containing glycerol); E, equilibrated (after 90 min of equilibration with glycerol at 4°C); T, thawed (after freezing–thawing); ALH, Amplitude of lateral head displacement (μm); BCF, Beat cross frequency (Hz); *within a step, indicates significant differences between treatments (*p* < 0.05). The values are represented as least square means ± SE.

SP only affected (*p* < 0.05) ALH and BCF in the last step (freezing–thawing) of the protocol (Figure 2). As may be observed, samples SP− exhibited higher (*p* < 0.05) values for ALH (2.29 μm) and BCF (11.5 Hz) after thawing than the samples SP+ (1.7 μm for ALH and 9.5 Hz for BCF).

**Figure 3:**
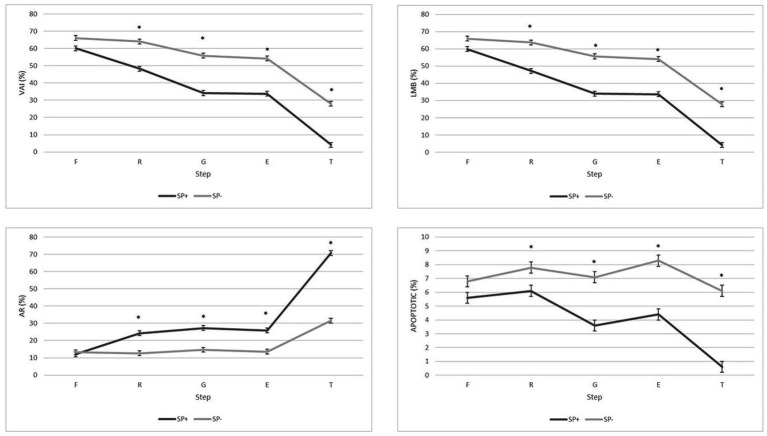
Goat bucks’ sperm quality (cytometer variables, *n* = 21) in each step of a freezing protocol when samples were processed in the presence (SP+) or in the absence (SP−) of SP. F, fresh (after centrifugation and addition of the first diluent); R, refrigerated (after reaching 4°C); G, glycerol (after addition of the extender containing glycerol); E, equilibrated (after 90 min of equilibration with glycerol at 4°C); T, thawed (after freezing–thawing); VAI, live sperm with intact acrosome (PI-/FITC-PNA-; %); LMB, live sperm with high mitochondrial membrane potential (PI−/Mitotracker+; %); AR, reacted acrosome sperm (FITC-PNA+;%); APOPTOTIC, apoptotic sperm (PI+ with low intensity; %); *within a step indicates significant differences between treatments (*p* < 0.05). The values of all the variables are represented as least square means ± SE.

The results for the variables analyzed in the flow cytometer are presented in Figure 3. Significant differences (*p* < 0.05) were observed between treatments except for the fresh step. The samples SP+ exhibited lower VAI, LMB, and APOPTOTIC values than the samples SP−. For the percentage of AR sperm, the samples SP+ exhibited higher percentages of AR sperm than the samples SP−.

Table 1 shows the differences between successive steps of the freezing protocol within each treatment (SP− and SP+). In both treatments, most differences appeared between the equilibration and the thawing steps (E-T column). In contrast, no differences were observed for any treatment between the glycerol addition and equilibrated steps (G-E column). The refrigeration (comparisons performed between the steps of fresh and refrigeration) was noxious for samples SP+ but not for samples SP−. The addition of glycerol mostly affected the percentages of TM sperm and the cytometer variables in both treatments, but a larger number of variables were modified in samples SP+ (R-G column).

**Table 1:** Effect of the different steps of the freezg protocol on goat buck’s sperm quality of samples SP+ or SP−.

	SP +				SP−			
Parameters	F-R	R-G	G-E	E-T	F-R	R-G	G-E	E-T
TM (%)	7.8 ± 3.7	17.8 ± 3.7*	−1.5 ± 3.7	45.0 ± 3.7*	−6.0 ± 3.7	14.3 ± 3.7*	−4.5 ± 3.7	38.9 ± 3.7*
PM (%)	−1.7 ± 3.8	15.1 ± 3.8*	−0.10 ± 3.8	37.3 ± 3.8*	−8.0 ± 3.8	10.2 ± 3.8	−3.8 ± 3.8	32.3 ± 3.8*
VSL (μm/s)	−9.5 ± 4.6	4.3 ± 4.7	0.17 ± 4.7	34.7 ± 4.6*	0.96 ± 4.6	2.2 ± 4.6	−1.4 ± 4.6	17.4 ± 4.6*
VAP (μm/s)	−8.3 ± 4.6	4.5 ± 4.7	0.20 ± 4.7	33.9 ± 4.6*	1.9 ± 4.6	2.9 ± 4.6	−2.0 ± 4.6	18.6 ± 4.6*
VCL (μm/s)	−6.3 ± 4.7	4.4 ± 4.7	0.22 ± 4.7	33.2 ± 4.7*	1.8 ± 4.7	4.1 ± 4.7	−1.5 ± 4.7	12.6 ± 4.7
LIN (%)	−8.8 ± 3.8	4.8 ± 3.8	−4.1 ± 3.8	11.2 ± 3.8	−2.1 ± 3.8	−1.8 ± 3.8	−1.2 ± 3.8	11.0 ± 3.8
STR (%)	−8.4 ± 4.2	5.6 ± 4.2	−5.1 ± 4.2	11.2 ± 4.2	−2.6 ± 4.2	−2.6 ± 4.2	0.9 ± 4.2	2.9 ± 4.2
WOB (%)	−6.6 ± 3.9	5.2 ± 3.9	−4.4 ± 3.9	9.0 ± 3.9	−0.4 ± 3.9	−1.4 ± 3.9	−2.0 ± 3.9	14.6 ± 3.9*
ALH (μm)	0.09 ± 0.09	0.07 ± 0.09	−0.06 ± 0.09	0.22 ± 0.09	−0.09 ± 0.09	0.09 ± 0.09	0.05 ± 0.09	−0.26 ± 0.09
BCF (Hz)	−0.35 ± 0.40	0.01 ± 0.41	0.05 ± 0.41	1.8 ± 0.40*	0.03 ± 0.40	0.05 ± 0.40	0.20 ± 0.40	−0.58 ± 0.40
VAI (%)	11.9 ± 2.0*	14.1 ± 2.0*	0.44 ± 2.0	29.6 ± 2.0*	1.9 ± 2.0	8.3 ± 2.0*	1.7 ± 2.0	26.2 ± 2.0*
LMB (%)	12.7 ± 2.0*	13.2 ± 2.0*	0.35 ± 2.0	29.5 ± 2.0*	2.2 ± 2.04	8.1 ± 2.0*	1.6 ± 2.0	26.2 ± 2.0*
AR (%)	−12.4 ± 1.9*	−3.1 ± 1.9	1.5 ± 1.9	−50.2 ± 1.9*	0.5 ± 1.9	−2.0 ± 1.9	1.1 ± 1.9	−18.4 ± 1.9*
APOPTOTIC (%)	−0.4 ± 0.5	2.4 ± 0.5*	−0.7 ± 0.5	3.7 ± 0.5*	−0.9 ± 0.5	0.7 ± 0.5	−1.3 ± 0.5	2.3 ± 0.5*
LIVE (%)	11.9 ± 2.0*	14.1 ± 2.0*	0.43 ± 2.0	29.6 ± 2.0*	1.9 ± 2.0	8.3 ± 2.0*	1.7 ± 2.0	26.2 ± 2.0*
MITOK (%)	15.0 ± 5.2	21.1 ± 5.2*	0.00 ± 5.2	21.0 ± 5.2*	0.2 ± 5.2	14.0 ± 5.2	2.4 ± 5.2	14.8 ± 5.2

Inside treatment (SP+ and SP−) comparisons were made between consecutive steps.

F, fresh (after centrifugation and addition of the first diluent); R, refrigerated (after reaching 4°C); G, glycerol (after addition of the extender containing glycerol); E, equilibrated (after 90 min of equilibration with glycerol at 4°C); T, thawed (after freezing–thawing); TM, total motile (%); PM, progressive motile (%); VSL, straight line velocity (μm/s); VAP, average path velocity (μm/s); VCL, curvilinear velocity (μm/s); LIN, linearity (%); STR, straightness index (%); WOB, wobble (%); ALH, Amplitude of lateral head displacement (μm); BCF, Beat cross frequency (Hz); VAI, live sperm with intact acrosome (PI-/FITC-PNA-; %); LMB, live sperm with high mitochondrial membrane potential (PI−/Mitotracker+;%); AR, reacted acrosome sperm (FITC-PNA+; %); APOPTOTIC: apoptotic sperm (PI+ with low intensity; %); LIVE, live sperm (PI-; plasma membrane intact sperm; %); MITOK, sperm with high mitochondrial membrane potential (Mitotracker+ with high intensity; %); * indicates significant differences between successive steps of the same treatment (*p* < 0.05). The values are represented as differences between steps for each measured parameter.

**Figure 4:**
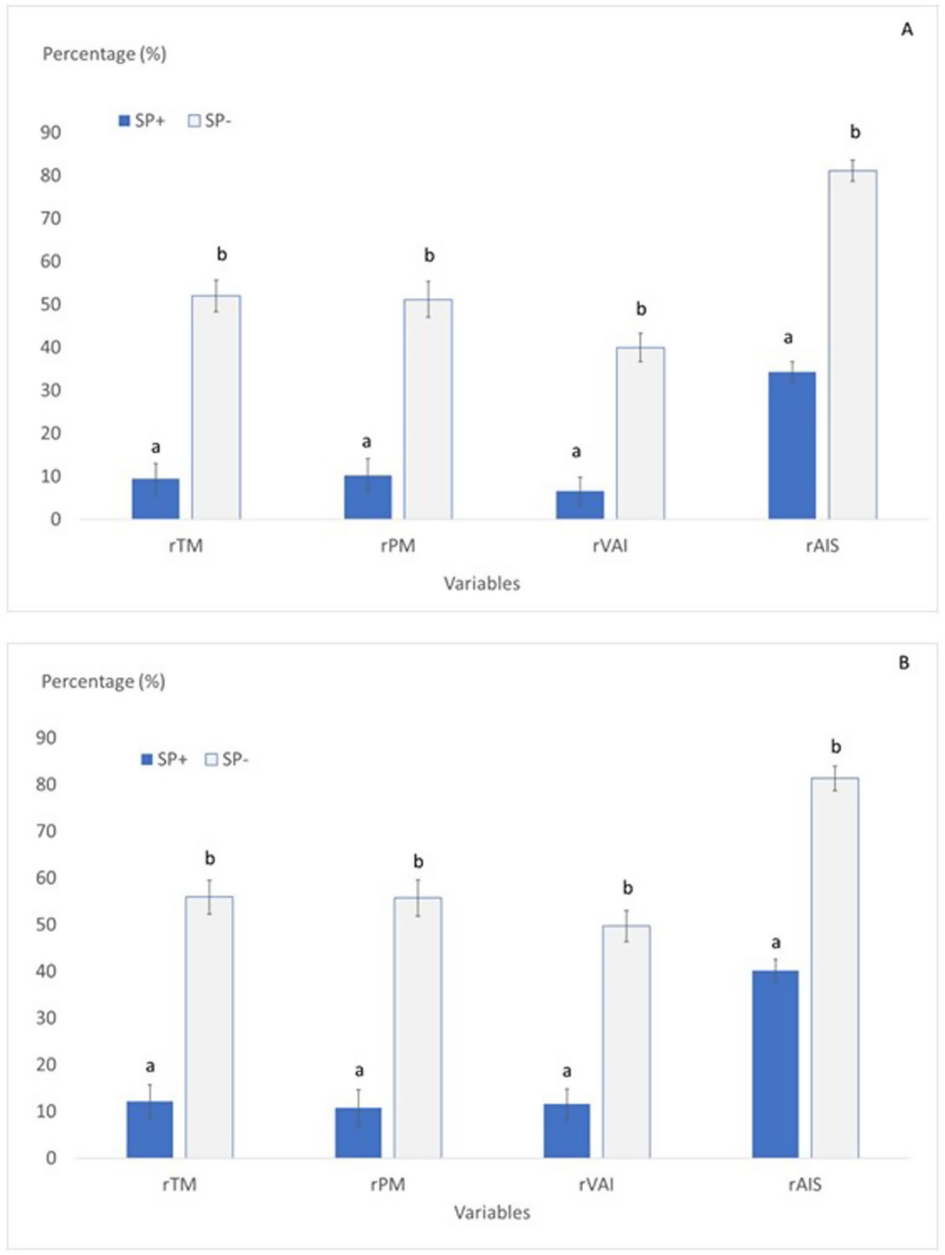
Relative changes observed between treatments (presence or absence of SP) in different steps of the process. Overall success [**(A)** comparisons between the steps of fresh and freezing–thawing] or success for the last step [**(B)** comparisons between the steps of equilibration and freezing–thawing] of the freezing protocol on goat buck’s sperm quality in samples processed in presence (SP+) or in absence (SP−) of SP. rTM (total motile sperm), calculated as follows: **(A)** rTM = (TM frozen–thawed/TM fresh) * 100 or **(B)** rTM = (TM frozen–thawed/TM equilibrated) * 100; rPM progressively motile sperm (%) calculated as follows: **(A)** rPM = (PM frozen–thawed/PM fresh) * 100 or **(B)** rPM = (PM frozen–thawed/PM equilibrated) * 100; rVAI: live acrosome intact sperm (PI-/FITC-PNA-; %) calculated as follows: **(A)** rVAI = (VAI frozen–thawed/ VAI fresh) * 100 or **(B)** rVAI = (VAI frozen–thawed/VAI equilibrated) * 100; rAIS: acrosome intact sperm (FITC-PNA-;%) calculated as follows: **(A)** rAIS = (AIS frozen–thawed/ AIS fresh) * 100 or (B) rAIS = (AIS frozen–thawed/AIS equilibrated) * 100. a, b: indicates significant differences (*p* < 0.05) between treatments (SP+ and SP−).

The elimination of SP was also clearly beneficial for the goat buck sperm quality (*p* < 0.05; Figure 4) for both, the success of the last step of the protocol (comparisons established between equilibrated and frozen–thawed sperm) and the success for all the process (comparisons established between fresh and frozen–thawed sperm).

For the overall success of the process (Figure 4A), the samples SP− retained after thawing between 40 and 52% of the values they exhibited in fresh for rTM, rPM or rVAI and near 80% of the acrosomes (rAIS), while these values dropped to 10% (between 6.6 and 10.2%) for rTM, rPM or rVAI and to 34% of rAIS sperm in the samples SP+.

Considering the success for the last step of the protocol (Figure 4B), the samples SP− retained 50% (between 50 and 56%) of the values they exhibited after equilibration for rTM, rPM or rVAI and near 80% of the sperm with the acrosome intact (rAIS), while in the samples SP+ these values dropped to 10% (between 11 and 12%) for rTM, rPM or rVAI and to 40% of rAIS sperm.

## 4 Discussion

Cryopreservation in liquid nitrogen preserves the fertilizing capacity of spermatozoa indefinitely, and *a priori*, these frozen semen doses should be a more attractive product for use in assisted reproductive techniques. However, the low fertility rates achieved with frozen goat semen present a significant obstacle to its use in commercial farms. In caprine, SP must be removed during the freezing process since there are interactions among some enzymes from the SP and some of the extender components [skimmed milk or egg yolk (10, 11)].

Although the SP of this species is considered an inadequate medium to maintain sperm viability after ejaculation (5) and its removal increases the percentage of viable cells and their motility during liquid storage (10), the presence of SP in refrigerated doses is not as noxious as it is for frozen semen. Thus, the damaging effect that SP has on the sperm is puzzling since it is observed in frozen semen (up to a point that the presence of SP is incompatible with sperm surviving the freezing–thawing process), but not in refrigerated semen, where SP is not removed. The most widely used protocol for SP removal before freezing consists of diluting the samples and centrifuging them to eliminate the supernatant containing the SP (9). The prejudicial effect of SP for frozen–thawed sperm is clear, but it is not known at which point of the freezing protocol the SP is lethal for the sperm. Identifying the point at which SP harms goat semen can help us improve freezing protocols, thereby increasing the quality and fertility rates of these doses.

Our motility and cytometry values for fresh semen SP+ fell within the range of the values published in other studies with the same breed [between 63 and 80% of TM and 57 and 68% VAI; (3, 17–21)]. On the other hand, the semen SP− also presented similar values to those observed in previous studies with the same breed and extender (20, 21).

According to our results, the noxious effect of SP begins to be observed in the refrigeration step of the protocol (Figures 1–3). After reaching 4°C (refrigeration), differences between treatments were observed for TM, PM, VAI, LMB, AR, and APOTOTIC. The values observed for the samples SP− indicate samples presenting higher sperm quality, except for the percentage of apoptotic spermatozoa, which was slightly higher for this treatment. Our results are within the range of values published in other studies with the same breed for the samples SP+ (3, 18, 19). Refrigeration induced more damage to the sperm SP+ than to the samples SP− (Table 1 column F-R). The worsening of the parameters VAI, LMB, LIVE, AR, and MITOK indicates that SP is especially noxious for the acrosome, plasma membrane, and sperm mitochondria. Surprisingly, the samples SP− had the same quality before and after refrigeration (Table 1 column F-R). The decrease in sperm quality after refrigeration is somehow expected, since when membranes are cooled, the phospholipids undergo a phase transition from a liquid state to the crystalline-gel state and the proteins cluster into the remaining liquid lipid domains which results in unstable membranes that have lost their functionality [reviewed by Mocé et al. (22)]. Therefore, upon rewarming, a decrease in sperm quality is normal.

The decrease in sperm quality after refrigeration in samples SP+ could be due to a direct or indirect effect of the lipases from the SP, since the enzymatic activity in samples SP+ cannot be discarded at these temperatures. Although the activity of BUSgp60 is higher at temperatures close to body temperature (23), enzymes are also functional at temperatures close to 0°C. However, the catalytic activity of mesophilic lipases decreases at these low temperatures (24). Therefore, BUSgp60 could act directly on sperm membrane and cause the hydrolysis of phospholipids, galactolipids, or triglycerides, or could degrade the SM residual triglycerides and produce oleic acid, which is toxic for the sperm (25). Oleic acid is known to damage the sperm membrane by increasing its lipid fusogenic activity or promoting premature acrosomal exocytosis, and it also negatively affects motility regulation via protein kinase C activation (25). These effects would explain the increase in the percentage of acrosome-reacted sperm observed in our study for the samples SP+.

The glycerol addition is fundamental in the freezing protocol since this is the step where the permeant cryoprotectant is added to the sperm. The addition of glycerol exerted negative effects in both treatments, since a drop in percentages of TM and cytometric parameters was observed compared to the values presented after refrigeration in samples SP−. This decrease in the percentage of total motile sperm is of the same order of magnitude as that observed in previous studies (9). Indeed, this decrease in sperm quality after adding the glycerol was expected, considering that SM2 is hyperosmolar and induces osmotic shock in the sperm. Glycerol is one of the most widely used cryoprotectants in diluents for goat sperm cryopreservation. However, it induces osmotic damage and may even be toxic to spermatozoa if added at high percentages. Although glycerol is permeant and traverses the membrane, it does so at a slower rate than water, so the sperm suffer from volume excursion [reviewed by Mocé et al. (22)]. Some authors pointed out that adding glycerol by itself may already cause some structural damage and provoke a decrease in sperm motility (26). However, the benefits of using glycerol in sperm cryopreservation surpass the disadvantages because glycerol contributes to the stabilization of lipid membranes, causing an increase in the fluidity of the sperm cell membrane under dehydrated conditions and therefore conferring the sperm greater capacity to survive cryopreservation (27).

The equilibration is necessary in the freezing process for changes to occur in the sperm membrane (28), and, according to our results, it does not add more damage or stress to the sperm. Thus, this step did not result in great differences from the previous step (glycerol addition) for any of the treatments, presenting the samples similar values after 90 min of equilibration with glycerol than immediately after its addition (Table 1).

The fact that the quality of the samples drops sharply after adding glycerol but not during the equilibration at 4°C for 90 min could indicate that the effect is likely more related to osmotic effects than any other cause in both treatments.

After freezing–thawing, differences between treatments were observed for all the variables except for VSL, VAP, LIN, STR, and WOB, which were similar for both treatments (Figures 1–3). Freezing–thawing was the step that resulted in more lethality for the sperm, irrespective of the presence or absence of SP. The freezing–thawing was more noxious for the samples SP+. Thus, when comparisons are made between the steps of equilibration and after freezing–thawing, the samples SP− retained around 50% (between 50 and 56%) of the values they exhibited after equilibration for TM, PM or VAI while in the samples SP+ these values dropped to 10% (between 11 and 12%; Figure 4B). These results were expected since freeze-thawing induces sperm damage and cryopreserved semen is always of lower quality than fresh semen (27). Also, the fact that samples SP− at the end of the procedure presented higher values than samples SP+ was expected and corroborates the results from previous studies (9). The sperm quality observed in this study for samples SP− was generally in close agreement with the reports from previous studies using the same extender (29, 30). This lethality may be attributed to the stresses the sperm must deal with during the freezing–thawing process that result from the interactions between water and solutes that occur through ice crystallization (27) and provoke osmotic shock. These osmotic effects provoke lethal or sublethal damage to the sperm, resulting in a decrease in sperm quality after freezing–thawing (27). Sperm cryopreservation is a complex process, and its major challenge is to cross the intermediate temperature zone between −15°C and −60°C. Sperm must pass through these temperatures twice, during freezing and thawing. Some authors point out that sperm injury during cryopreservation is more related to the negative effect of thawing (31). Although in this step of the protocol most of the damage was expected to be attributed to the stresses the sperm suffer because of the ice crystals formation and the characteristics of the remaining unfrozen fraction, the SP and/or the oleic acid that may have been released in previous steps still contribute to increase the damage during this step in samples SP+ since in relative terms the drop in quality was double in these samples than in the samples SP−. Oleic acid alters the structure, fluidity, and permeability of phospholipid monolayers and bilayers (32). If the oleic acid has modified the sperm membrane permeability in the samples SP+, the sperm ability to adapt to the osmotic changes that occur during the cryopreservation process will also be altered.

The movement kinetics from motile thawed sperm differ from those observed before freezing, since these sperm exhibited slower velocities than the motile sperm before freezing in both treatments. After thawing, ALH was significantly higher in the samples SP- than in the samples SP+. Some studies linked elevated values of this parameter with premature sperm capacitation since an increase in the amplitude of lateral head displacement is observed in hyperactivation motility patterns (33). These changes in motility kinetics probably manifest the phenomenon coined as cryocapacitation that renders sperm with a short lifespan for use *in vivo* (34).

Considering all the processes (from fresh to freeze-thawing), the success for samples in which SP was eliminated was that 40% of the values exhibited by VAI and 52% of the values exhibited by TM in fresh were retained after thawing. The relative retention of TM and PM after all the processes for the samples SP− is like that obtained in previous studies with goat buck sperm (35). This result is considered normal since cryopreservation is lethal for circa 50% of the sperm in a typical semen sample (27). However, the cryopreservation process was unsuccessful for the samples frozen with the SP since these samples only retained 9.4% for TM and 6.6% for VAI of the values they presented in fresh.

## 5 Conclusion

In conclusion, the presence of SP was detrimental to the survival of goat buck sperm since the refrigeration step, although most of the damage was inflicted during the freeze-thawing step. Since the equilibration step does not impact sperm quality, resources and efforts should be reallocated to enhance the results in the other steps that significantly affect sperm quality. The detrimental effects of freezing–thawing suggest that this step should be the primary focus of improvement efforts in sperm cryopreservation protocols. Moreover, the negative effects of SP, evident from the refrigeration step, highlight the importance of developing early intervention strategies to minimize damage before the sperm undergoes freezing.

## Data Availability

The raw data supporting the conclusions of this article will be made available by the authors without undue reservation.
